# Demonstration of cooling by the Muon Ionization Cooling Experiment

**DOI:** 10.1038/s41586-020-1958-9

**Published:** 2020-02-05

**Authors:** MICE collaboration, M. Bogomilov, R. Tsenov, G. Vankova-Kirilova, Y. P. Song, J. Y. Tang, Z. H. Li, R. Bertoni, M. Bonesini, F. Chignoli, R. Mazza, V. Palladino, A. de Bari, D. Orestano, L. Tortora, Y. Kuno, H. Sakamoto, A. Sato, S. Ishimoto, M. Chung, C. K. Sung, F. Filthaut, D. Jokovic, D. Maletic, M. Savic, N. Jovancevic, J. Nikolov, M. Vretenar, S. Ramberger, R. Asfandiyarov, A. Blondel, F. Drielsma, Y. Karadzhov, S. Boyd, J. R. Greis, T. Lord, C. Pidcott, I. Taylor, G. Charnley, N. Collomb, K. Dumbell, A. Gallagher, A. Grant, S. Griffiths, T. Hartnett, B. Martlew, A. Moss, A. Muir, I. Mullacrane, A. Oates, P. Owens, G. Stokes, P. Warburton, C. White, D. Adams, V. Bayliss, J. Boehm, T. W. Bradshaw, C. Brown, M. Courthold, J. Govans, M. Hills, J.-B. Lagrange, C. Macwaters, A. Nichols, R. Preece, S. Ricciardi, C. Rogers, T. Stanley, J. Tarrant, M. Tucker, S. Watson, A. Wilson, R. Bayes, J. C. Nugent, F. J. P. Soler, G. T. Chatzitheodoridis, A. J. Dick, K. Ronald, C. G. Whyte, A. R. Young, R. Gamet, P. Cooke, V. J. Blackmore, D. Colling, A. Dobbs, P. Dornan, P. Franchini, C. Hunt, P. B. Jurj, A. Kurup, K. Long, J. Martyniak, S. Middleton, J. Pasternak, M. A. Uchida, J. H. Cobb, C. N. Booth, P. Hodgson, J. Langlands, E. Overton, V. Pec, P. J. Smith, S. Wilbur, M. Ellis, R. B. S. Gardener, P. Kyberd, J. J. Nebrensky, A. DeMello, S. Gourlay, A. Lambert, D. Li, T. Luo, S. Prestemon, S. Virostek, M. Palmer, H. Witte, D. Adey, A. D. Bross, D. Bowring, A. Liu, D. Neuffer, M. Popovic, P. Rubinov, B. Freemire, P. Hanlet, D. M. Kaplan, T. A. Mohayai, D. Rajaram, P. Snopok, Y. Torun, L. M. Cremaldi, D. A. Sanders, D. J. Summers, L. R. Coney, G. G. Hanson, C. Heidt

**Affiliations:** 1 Department of Atomic Physics, St Kliment Ohridski University of Sofia, Sofia, Bulgaria; 2 Institute of High Energy Physics, Chinese Academy of Sciences, Beijing, China; 3 Sichuan University, Chengdu, China; 4 Sezione INFN Milano Bicocca, Dipartimento di Fisica G. Occhialini, Milan, Italy; 5 Sezione INFN Napoli and Dipartimento di Fisica, Università Federico II, Complesso Universitario di Monte S. Angelo, Naples, Italy; 6 Sezione INFN Pavia and Dipartimento di Fisica, Pavia, Italy; 7 INFN Sezione di Roma Tre and Dipartimento di Matematica e Fisica, Università Roma Tre, Rome, Italy; 8 Osaka University, Graduate School of Science, Department of Physics, Toyonaka, Japan; 9 High Energy Accelerator Research Organization (KEK), Institute of Particle and Nuclear Studies, Tsukuba, Japan; 10 UNIST, Ulsan, South Korea; 11 Nikhef, Amsterdam, The Netherlands; 12 Radboud University, Nijmegen, The Netherlands; 13 Institute of Physics, University of Belgrade, Belgrade, Serbia; 14 Faculty of Sciences, University of Novi Sad, Novi Sad, Serbia; 15 CERN, Geneva, Switzerland; 16 DPNC, Section de Physique, Université de Genève, Geneva, Switzerland; 17 Department of Physics, University of Warwick, Coventry, UK; 18 STFC Daresbury Laboratory, Daresbury, Cheshire, UK; 19 STFC Rutherford Appleton Laboratory, Harwell Oxford, Didcot, UK; 20 Brunel University, Uxbridge, UK; 21 School of Physics and Astronomy, The University of Glasgow, Glasgow, UK; 22 SUPA and Department of Physics, University of Strathclyde, Glasgow, UK; 23 Cockcroft Institute, Daresbury Laboratory, Daresbury, UK; 24 Department of Physics, University of Liverpool, Liverpool, UK; 25 Department of Physics, Blackett Laboratory, Imperial College London, London, UK; 26 Department of Physics, University of Oxford, Oxford, UK; 27 Department of Physics and Astronomy, University of Sheffield, Sheffield, UK; 28 Lawrence Berkeley National Laboratory, Berkeley, CA USA; 29 Brookhaven National Laboratory, Upton, NY USA; 30 Fermilab, Batavia, IL USA; 31 Illinois Institute of Technology, Chicago, IL USA; 32 University of Mississippi, Oxford, MS USA; 33 University of California, Riverside, CA USA; 34 Present Address: RIKEN 2-1 Horosawa, Wako, Japan; 35 Present Address: Department of Physics and Astronomy, University of Sheffield, Sheffield, UK; 36 Present Address: Defence Science and Technology Laboratory, Salisbury, UK; 37 Present Address: ATC, Royal Observatory Edinburgh, Edinburgh, UK; 38 Present Address: Laurentian University, Sudbury, Ontario Canada; 39 Present Address: OPERA Simulation Software, Kidlington, UK; 40 Present Address: CERN, Geneva, Switzerland; 41 Present Address: School of Physics and Astronomy, University of Manchester, Manchester, UK; 42 Present Address: Cavendish Laboratory, Cambridge, UK; 43 Present Address: Arm, Sheffield, UK; 44 Present Address: Westpac Group, Sydney, New South Wales Australia; 45 Present Address: Institute of High Energy Physics, Chinese Academy of Sciences, Bejing, China; 46 Present Address: Euclid Techlabs, Bolingbrook, IL USA; 47 Present Address: Fermilab, Batavia, IL USA; 48 Present Address: European Spallation Source ERIC, Lund, Sweden

**Keywords:** Mechanical engineering, Experimental nuclear physics, Experimental particle physics

## Abstract

The use of accelerated beams of electrons, protons or ions has furthered the development of nearly every scientific discipline. However, high-energy muon beams of equivalent quality have not yet been delivered. Muon beams can be created through the decay of pions produced by the interaction of a proton beam with a target. Such ‘tertiary’ beams have much lower brightness than those created by accelerating electrons, protons or ions. High-brightness muon beams comparable to those produced by state-of-the-art electron, proton and ion accelerators could facilitate the study of lepton–antilepton collisions at extremely high energies and provide well characterized neutrino beams^1–6^. Such muon beams could be realized using ionization cooling, which has been proposed to increase muon-beam brightness^7,8^. Here we report the realization of ionization cooling, which was confirmed by the observation of an increased number of low-amplitude muons after passage of the muon beam through an absorber, as well as an increase in the corresponding phase-space density. The simulated performance of the ionization cooling system is consistent with the measured data, validating designs of the ionization cooling channel in which the cooling process is repeated to produce a substantial cooling effect^9–11^. The results presented here are an important step towards achieving the muon-beam quality required to search for phenomena at energy scales beyond the reach of the Large Hadron Collider at a facility of equivalent or reduced footprint^6^.

## Abstract (web summary)

Ionization cooling, a technique that delivers high-brightness muon beams for the study of phenomena at energy scales beyond those of the Large Hadron Collider, is demonstrated by the Muon Ionization Cooling Experiment.

## High-quality muon beams

Fundamental insights into the structure of matter and the nature of its elementary constituents have been obtained using beams of charged particles. The use of time-varying electromagnetic fields to produce sustained acceleration was pioneered in the 1930s^12–14^. Since then, high-energy and high-brightness particle accelerators have delivered electron, proton and ion beams for applications ranging from the search for new phenomena in the interactions of quarks and leptons to the study of nuclear physics, materials science and biology.

Muon beams can be created using a proton beam striking a target to produce a secondary beam comprising many particle species including pions, kaons and muons. The pions and kaons decay to produce additional muons, which are captured by electromagnetic beamline elements to produce a tertiary muon beam. Capture must be realized on a timescale compatible with the muon lifetime at rest, 2.2 μs. Without acceleration, the energy and intensity of the muon beam is limited by the energy and intensity of the primary proton beam and the efficiency with which muons are captured.

Accelerated high-brightness muon beams have been proposed as a source of neutrinos at neutrino factories and for the delivery of multi-TeV lepton–antilepton collisions at muon colliders^1–6^. Muons have attractive properties for the delivery of high-energy collisions. The muon is a fundamental particle with mass 207 times that of the electron. This high mass results in suppression of synchrotron radiation, potentially enabling collisions between beams of muons and antimuons at energies far in excess of those that can be achieved in an electron–positron collider, such as the proposed International Linear Collider^15^, the Compact Linear Collider^16^, the Circular Electron–Positron Collider^17^ and the electron–positron option of the Future Circular Collider^18^. The virtual absence of synchrotron radiation makes it possible to build a substantially smaller facility with the same or greater physics reach.

The energy available in collisions between the constituent gluons and quarks in proton–proton collisions is considerably less than the energy of the proton beam because the colliding quarks and gluons each carry only a fraction of the proton’s momentum. Muons carry the full energy of the beam, making muon colliders attractive for the study of particle physics beyond the energy reach of facilities such as the Large Hadron Collider^19^.

Most of the proposals for accelerated muon beams exploit the proton-driven muon-beam production scheme outlined above and use beam cooling to increase the brightness of the tertiary muon beam before acceleration and storage to ensure sufficient luminosity or beam current. Four cooling techniques are in use at particle accelerators: synchrotron radiation cooling^20^, laser cooling^21^, stochastic cooling^22^ and electron cooling^23^. In each case, the time required to cool the beam is long compared to the muon lifetime. Frictional cooling of muons, in which muons are electrostatically accelerated through an energy-absorbing medium at energies significantly below 1 MeV, has been demonstrated but with low efficiency^24–26^.

The technique demonstrated in this study, ionization cooling^7,8^, is based on a suitably prepared beam passing through an appropriate material (the absorber) and losing momentum through ionization. Radio-frequency cavities restore momentum only along the beam direction. Passing the muon beam through a repeating lattice of material and accelerators causes the ionization cooling effect to build up in a time much shorter than the muon lifetime^9–11^. Acceleration of a muon beam in a radio-frequency accelerator has recently been demonstrated^27^, and reduced beam heating, damped by the ionization cooling effect, has been observed^28^. Ionization cooling has not been demonstrated so far. Experimental validation of the technique is important for the development of muon accelerators. The international Muon Ionization Cooling Experiment (MICE; http://mice.iit.edu) was designed to demonstrate transverse ionization cooling, the realization of which is presented here.

The brightness of a particle beam can be characterized by the number of particles in the beam and the volume occupied by the beam in position–momentum phase space. The phase-space volume occupied by the beam and the phase-space density of the beam are conserved quantities in a conventional accelerator without cooling. The phase space considered here is the position and momentum transverse to the direction of travel of the beam, **u** = (*x*, *p*_*x*_, *y*, *p*_*y*_), where *x* and *y* are coordinates perpendicular to the beam line, and *p*_*x*_ and *p*_*y*_ are the corresponding components of the momentum. The *z* axis is the nominal beam axis.

The normalized root-mean-square (r.m.s.) emittance is conventionally used as an indicator of the phase-space volume occupied by the beam^29^, but this quantity is not conserved when scraping or optical aberrations affect the edge of the beam. The distribution of amplitudes^30,31^ is used here to study effects in the core of the beam. The amplitude of a particle is the distance of the particle from the beam centroid in normalized phase space, and is a conserved quantity in a conventional accelerator without cooling. The phase-space density of the beam is also directly studied using a *k*-nearest-neighbour technique^32^.

## MICE cooling apparatus

**Fig. 1:**
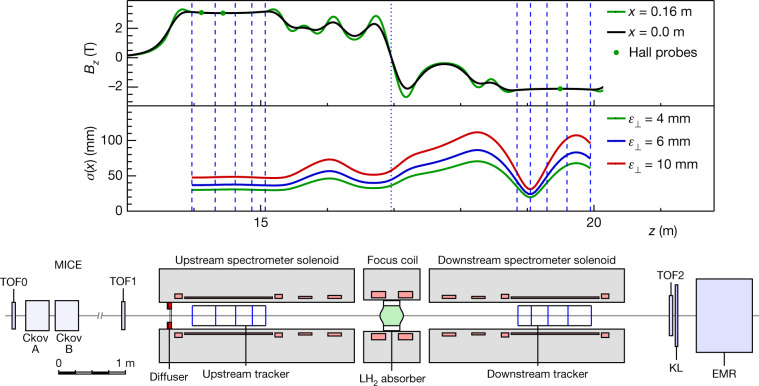
The MICE apparatus, the calculated magnetic field and the nominal horizontal width of the beam. The modelled field, *B*_*z*_, is shown on the beam axis (black line) and at 160 mm from the axis (green line) in the horizontal plane. The readings of Hall probes situated at 160 mm from the beam axis are also shown. Vertical lines indicate the positions of the tracker stations (dashed lines) and the absorber (dotted line). The nominal r.m.s. beam width, *σ*(*x*), is calculated assuming a nominal input beam and using linear beam transport equations. See text for the description of the MICE apparatus. TOF0, TOF1 and TOF2 are time-of-flight detector stations; KL is a lead–scintillator pre-shower detector; EMR is the Electron–Muon Ranger.

The MICE collaboration has built a tightly focusing solenoid lattice, absorbers and instrumentation to demonstrate the ionization cooling of muons. A schematic of the apparatus is shown in Fig. 1.

A transfer line^33–35^ brought a beam, composed mostly of muons, from a target^36^ in the ISIS synchrotron^37^ to the cooling apparatus. The central momentum of the muons could be tuned between 140 MeV *c*^−1^ and 240 MeV *c*^−1^ (*c*, speed of light in vacuum). A variable-thickness brass and tungsten diffuser allowed the emittance of the incident beam to be varied between 4 mm and 10 mm.

The tight focusing (low β function) and large acceptance required by the cooling section was achieved using 12 superconducting solenoids. The solenoids were contained in three warm-bore modules cooled by closed-cycle cryocoolers. The upstream and downstream modules (spectrometer solenoids) were identical, each containing three coils to provide a uniform field region of up to 4 T within the 400-mm-diameter warm bore for momentum measurement, as well as two ‘matching’ coils to match the beam to the central pair of closely spaced ‘focus’ coils, which focus the beam onto the absorber. The focus coils were designed to enable peak on-axis fields of up to 3.5 T within one module with a 500-mm-diameter warm bore containing the absorbers.

For the experiment reported here the focus coils were operated in ‘flip’ mode with a field reversal at the centre. Because the magnetic lattice was tightly coupled, the cold mass-suspension systems of the modules were designed to withstand longitudinal cold-to-warm forces of several hundred kN, which could arise during an unbalanced quench of the system. At maximum field, the inter-coil force on the focus coil cold mass was of the order of 2 MN. The total energy stored in the magnetic system was of the order of 5 MJ and the system was protected by both active and passive quench-protection systems. The normal charging and discharging time of the solenoids was several hours. The entire magnetic channel was partially enclosed by a 150-mm-thick soft-iron return yoke for external magnetic shielding. The magnetic fields in the tracking volumes were monitored during operation using calibrated Hall probes.

One of the matching coils in the downstream spectrometer solenoid was not operable owing to a failure of a superconducting lead. Although this necessitated a compromise in the lattice optics and acceptance, the flexibility of the magnetic lattice was exploited to ensure a clear cooling measurement.

The amplitude acceptance of approximately 30 mm, above which particles scrape, was large compared to that of a typical accelerator. Even so, considerable scraping was expected and observed for the highest-emittance beams. Ionization cooling cells with even larger acceptances, producing less scraping, have been designed^9–11^. The magnetic lattice of MICE, shown in Fig. 1, was tuned so that the focus of the beam was near the absorber, resulting in a small beam width and large angular divergence. The tight focusing, corresponding to a nominal transverse β function of around 430 mm at the centre of the absorber, yielded an optimal cooling performance.

Materials with low atomic number, such as lithium and hydrogen, have a long radiation length relative to the rate of energy loss, and consequently better cooling performance, making them ideal absorber materials. Therefore, cooling by both liquid-hydrogen and lithium hydride absorbers was studied.

The liquid hydrogen was contained within a 22-l vessel^38^ in the warm bore of the focus coil. Hydrogen was liquefied by a cryocooler and piped through the focus coil module into the absorber body. When filled, the absorber presented 349.6 ± 0.2 mm of liquid hydrogen along the beam axis with a density of 0.07053 ± 0.00008 g cm^−3^ (all uncertainties represent the standard error). The liquid hydrogen was contained between a pair of aluminium windows covered by multi-layer insulation. A second pair of windows provided a secondary barrier to protect against failure of the primary containment windows. These windows were designed to be as thin as possible so that any scattering in them would not cause substantial heating. The total thickness of all four windows on the beam axis was 0.79 ± 0.01 mm.

The lithium hydride absorber was a disk of thickness 65.37 ± 0.02 mm with a density of 0.6957 ± 0.0006 g cm^−3^. The isotopic composition of the lithium used to produce the absorber was 95% ^6^Li and 5% ^7^Li. The cylinder had a thin coating of parylene to prevent ingress of water or oxygen. Configurations with the empty liquid-hydrogen containment vessel and with no absorber were also studied.

## MICE beam instrumentation

Detectors placed upstream and downstream of the apparatus measured the momentum, position and species of each particle entering and leaving the cooling channel in order to reconstruct the full four-dimensional phase space, including the angular momentum introduced by the solenoids. Particles were recorded by the apparatus one at a time, which enabled high-precision instrumentation to be used and particles other than muons to be excluded from the analysis. Each ensemble of muons was accumulated over a number of hours. This is acceptable because space-charge effects are not expected at a neutrino factory and in a muon collider they become important only at very low longitudinal emittance^39^. Data-taking periods for each absorber were separated by a period of weeks owing to operational practicalities. The phase-space distribution of the resulting ensemble was reconstructed using the upstream and downstream detectors. The emittance reconstruction in the upstream detector system is described in ref. ^40^.

Upstream of the cooling apparatus, two time-of-flight (TOF) detectors^41^ measured the particle velocity. A complementary velocity measurement was made upstream by the threshold Cherenkov counters Ckov A and Ckov B^42^. Scintillating fibre trackers, positioned in the uniform-field region of each of the two spectrometer solenoids, measured the particle position and momentum upstream and downstream of the absorber^43,44^. Downstream, an additional TOF detector^45^, a mixed lead–scintillator pre-shower detector and a totally active scintillator calorimeter, the Electron–Muon Ranger^46,47^, identified electrons produced by muon decay and allowed cross-validation of the measurements made by the upstream detectors and the trackers.

Each tracker consisted of five planar scintillating-fibre stations. Each station comprised three views; each view was composed of two layers of 350-μm-diameter scintillating fibres positioned at an angle of 120° with respect to the other views. The fibres were read out by cryogenic visible-light photon counters^48^. The position of a particle crossing the tracker was inferred from the coincidence of signals from the fibres, and the momentum was calculated by fitting a helical trajectory to the signal positions, with appropriate consideration for energy loss and scattering in the fibres.

Each TOF detector was constructed from two orthogonal planes of scintillator slabs. Photomultiplier tubes at each end of every TOF detector slab were used to determine the time at which a muon passed through the apparatus with a 60-ps resolution^41^. The momentum resolution of particles with a small helix radius in the tracker was improved by combining the TOF measurement of velocity with the measurement of momentum in the tracker.

A detailed Monte Carlo simulation of the experiment was performed to study the resolution and efficiency of the instrumentation and to determine the expected performance of the cooling apparatus^49,50^. The simulation was found to give a good description of the data^40^.

## Demonstration of cooling

**Fig. 2:**
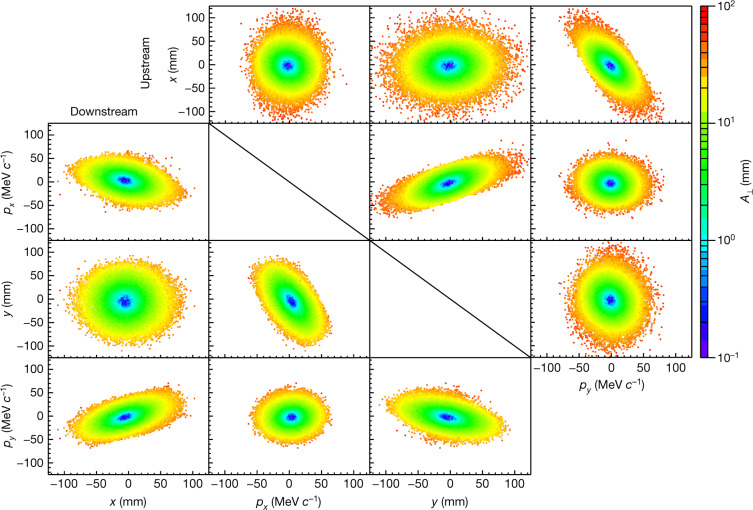
Beam distribution in phase space for the 6–140 Full LH2 setting of MICE. Measured beam distribution in the upstream tracker (above the diagonal) and in the downstream tracker (below the diagonal). The measured coordinates of the particles are coloured according to the amplitude *A*_⊥_ of the particle.

The data presented here were taken using beams with a nominal momentum of 140 MeV *c*^−1^ and a nominal normalized r.m.s. emittance in the upstream tracking volume of 4 mm, 6 mm and 10 mm; these settings are denoted as ‘4–140’, ‘6–140’ and ‘10–140’, respectively. Beams with a higher emittance have more muons at high amplitudes and occupy a larger region in phase space. For each beam setting, two samples were considered for the analysis. The ‘upstream sample’ contained particles identified as muons by the upstream TOF detectors and tracker, for which the muon trajectory reconstructed in the upstream tracker was fully contained in the fiducial volume and for which the reconstructed momentum fell within the range 135 MeV *c*^−1^ to 145 MeV *c*^−1^ (which is considerably higher than the momentum resolution of the tracker, 2 MeV *c*^−1^). The ‘downstream sample’ was the subset of the upstream sample for which the reconstructed muons were fully contained in the fiducial volume of the downstream tracker. Each of the samples had between 30,000 and 170,000 events. Examples of the phase-space distributions of the particles in the two samples are shown in Fig. 2. The strong correlations between *y* and *p*_*x*_ and between *x* and *p*_*y*_ are due to the angular momentum introduced by the solenoidal field. The shorter tails along the semi-minor axis compared to the semi-major axis in these projections arise from scraping in the diffuser.

**Fig. 3:**
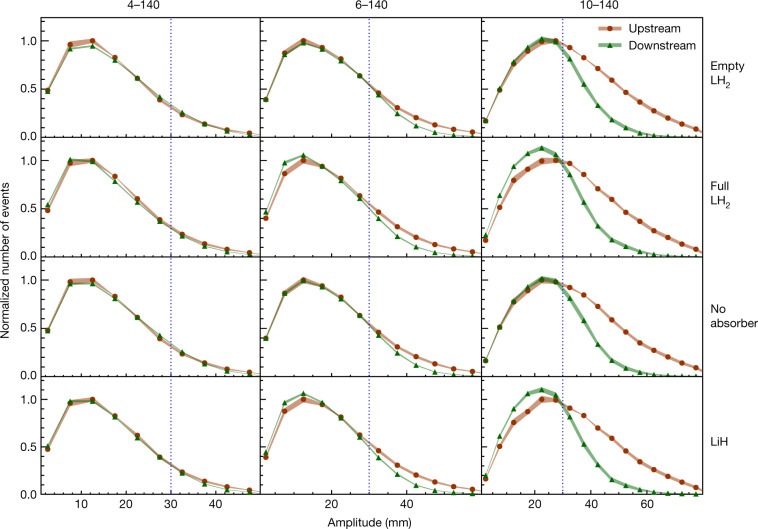
Muon amplitudes measured by MICE. The measured upstream distributions are shown by red circles while the downstream distributions are shown by green triangles. Both upstream and downstream distributions are normalized to the bin with the most entries in the upstream distribution (see text). Coloured bands show the estimated standard error, which is dominated by systematic uncertainties. Vertical lines indicate the approximate channel acceptance above which scraping occurs. The number of events in each sample is listed in Extended Data Table 2. Data for each experimental configuration were accumulated in a single discrete period. Source Data

The distributions of amplitudes in the upstream and downstream samples for each of the 4–140, 6–140 and 10–140 datasets are shown in Fig. 3. The nominal acceptance of the magnetic channel is also indicated. A correction has been made to account for the migration of events between amplitude bins due to the detector resolution and to account for inefficiency in the downstream detector system (see Methods). Distributions are shown for the measurements with an empty liquid-hydrogen vessel (‘Empty LH_2_’), with a filled liquid-hydrogen vessel (‘Full LH_2_’), with no absorber (‘No absorber’) and with the lithium hydride absorber (‘LiH’). The distributions were normalized to allow a comparison of the shape of the distribution between different absorbers. Each pair of upstream and downstream amplitude distributions is scaled by $1/{N}_{\max }^{{\rm{u}}}$, where ${N}_{\max }^{{\rm{u}}}$ is the number of events in the most populated bin in the upstream sample.

The behaviour of the beam at low amplitude is the key result of this study. For the ‘No absorber’ and ‘Empty LH_2_’ configurations, the number of events with low amplitude in the downstream sample is similar to that observed in the upstream sample. For the 6–140 and 10–140 configurations for both the ‘Full LH_2_’ and the ‘LiH’ samples, the number of events with low amplitude is considerably larger in the downstream sample than in the upstream sample. This indicates an increase in the number of particles in the beam core when an absorber is installed, which is expected if ionization cooling takes place. This effect can occur only because energy loss is a non-conservative process.

A reduction in the number of muons at high amplitude is also observed, especially for the 10–140 setting. Whereas part of this effect arises owing to migration of muons into the beam core, a substantial number of high-amplitude particles outside the beam acceptance intersected the beam pipe or fell outside the fiducial volume of the downstream tracker. The beam pipe was made of materials with higher atomic number than those of the absorber materials, so interactions in the beam pipe tended to be dominated by multiple Coulomb scattering, leading to beam loss.

A *χ*^2^ test was performed to determine the confidence with which the null hypothesis that for the same input beam setting, the amplitude distributions in the downstream samples of the ‘Full LH_2_’ and ‘Empty LH_2_’ configurations are compatible, and the amplitude distributions in the downstream samples of the ‘LiH’ and ‘No absorber’ configurations are compatible. The test was performed on the uncorrected distributions using only statistical uncertainties. Systematic effects are the same for the pairs of distributions tested, and cancel. Assuming that this null hypothesis is correct, the probability of observing the effect seen in the data is considerably lower than 10^−5^ for each beam setting and for each ‘Full LH_2_’–‘Empty LH_2_’ and ‘LiH’–‘No absorber’ pair; therefore, the null hypothesis was rejected.

The fractional increase in the number of particles with low amplitude is most pronounced for the 10–140 beams. High-amplitude beams have high transverse emittance, *ε*_⊥_, and a larger transverse momentum relative to the stochastic increase in transverse momentum due to scattering, so they undergo more cooling. For the magnet settings and beams studied here, heating due to multiple Coulomb scattering becomes dominant over ionization cooling at an emittance of around 4 mm. As a result, only modest cooling is observed for the 4–140 setting in both the ‘Full LH_2_’ and ‘LiH’ configurations.

**Fig. 4:**
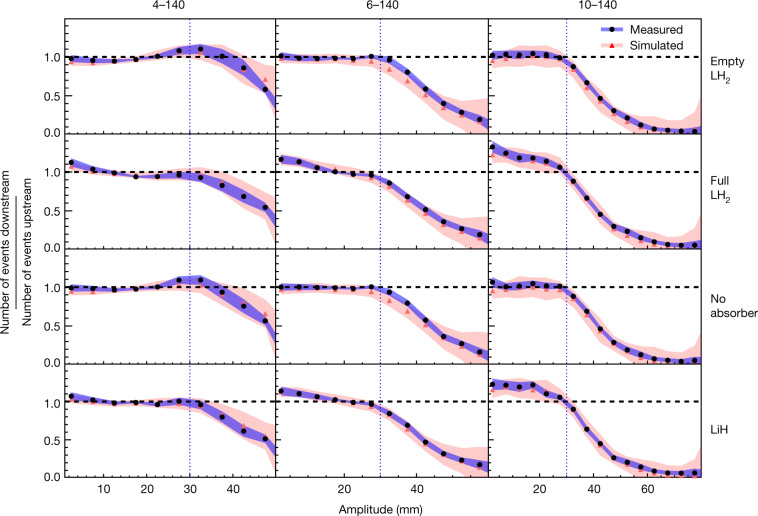
Downstream-to-upstream ratio of number of events in MICE. A ratio greater than unity in the beam core, which is evidence of ionization cooling, is observed in the data obtained with the 6–140 and 10–140 beams with both the full LH_2_ absorber and the LiH absorber. The effect predicted from the simulation is shown in red and that measured is shown in black. The corresponding shading shows the estimated standard error, which is dominated by systematic uncertainty. Vertical lines indicate the channel acceptance above which scraping occurs. The number of events in each sample is listed in Extended Data Table 2. Data for each experimental configuration were accumulated in a single discrete period. Source Data

The ratios of the downstream to the upstream amplitude distributions are shown in Fig. 4. In the ‘No absorber’ and ‘Empty absorber’ configurations, the ratios are consistent with 1 for amplitudes of less than 30 mm, confirming the conservation of amplitude in this region, irrespective of the incident beam. Above 30 mm the ratios drop below unity, indicating that at high amplitude there are fewer muons downstream than upstream, as outlined above. The presence of the absorber windows does not strongly affect the amplitude distribution. For the 6–140 and 10–140 datasets, the addition of liquid-hydrogen or lithium hydride absorber material causes the ratios to rise above unity for the low-amplitude particles that correspond to the beam core. This indicates an increase in the number of particles in the beam core and demonstrates ionization cooling.

**Fig. 5:**
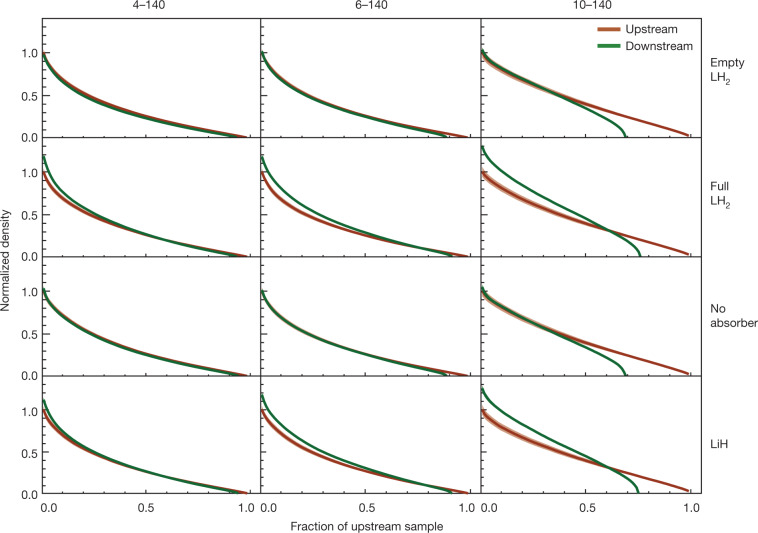
Normalized quantile distribution of the beam density in MICE. Upstream and downstream quantiles are indicated by orange and green lines, respectively, as a function of the fraction of the upstream sample. For each configuration, the density is normalized to the highest-density region in the upstream sample. The estimated standard error is indicated by the thickness of the coloured bands and is dominated by systematic uncertainty. The number of events in each sample is listed in Extended Data Table 2. Data for each experimental configuration were accumulated in a single discrete period. Source Data

The density in phase space is an invariant of a symplectic system; therefore, an increase in phase-space density is also an unequivocal demonstration of cooling. Figure 5 shows the normalized density of the upstream and downstream samples, *ρ*_*i*_(**u**_*i*_)/*ρ*_0_, as a function of *α*, the fraction of the upstream sample that has a density greater than or equal to *ρ*_*i*_. This is known as the quantile distribution. To enable comparison between different beam configurations, the densities for each configuration have been normalized to the peak density in the upstream tracker, *ρ*_0_. To enable comparison between the upstream and downstream distributions, the fraction of the sample is always relative to the total number of events in the upstream sample. The transmission is the fraction of the beam for which the density in the downstream tracker reaches zero. For the ‘No absorber’ and ‘Empty LH_2_’ cases, the downstream density in the highest-density regions is indistinguishable from the upstream density. A small amount of scraping is observed for the 4–140 and 6–140 beams. More substantial scraping is observed for the 10–140 beam. In all cases, for ‘Full LH_2_’ and ‘LiH’ the phase-space density increases, and the increase is greater for higher-emittance beams. These observations demonstrate the ionization cooling of the beam when an absorber is installed. In the presence of an absorber, beams with larger nominal emittance show a greater increase in density than those with a lower nominal emittance.

## Conclusions

Ionization cooling has been unequivocally demonstrated. We have built and operated a section of a solenoidal cooling channel and demonstrated the ionization cooling of muons using both liquid hydrogen and lithium hydride absorbers. The effect has been observed through the measurement of both an increase in the number of small-amplitude particles (Figs. 3, 4) and an increase in the phase-space density of the beam (Fig. 5). The results are well described by simulations (Fig. 4). This demonstration of ionization cooling is an important advance in the development of high-brightness muon beams. The seminal results presented in this paper encourage further development of high-brightness muon beams as a tool for the investigation of the fundamental properties of matter.

## Methods

### Characterization of beam brightness

In particle accelerators, the average beam brightness $\mathop{B}\limits^{-}$ is defined as the beam current, *I*, passing through a transverse phase-space volume ${{\mathscr{V}}}_{4}$ (ref. ^51^)

1 $$\mathop{B}\limits^{-}=\frac{I}{{{\mathscr{V}}}_{4}}$$

The normalized r.m.s. emittance is often used as an indicator of the phase-space volume occupied by the beam and is given by^29^

2 $${\varepsilon }_{\perp }=\frac{\sqrt[4]{|{V}|}}{{m}_{\mu }c}$$

where *m*_*μ*_ is the muon mass and |*V*| is the determinant of the covariance matrix of the beam in the transverse phase space **u** = (*x*, *p*_*x*_, *y*, *p*_*y*_). The covariance matrix has elements ${v}_{ij}=\langle {u}_{i}{u}_{j}\rangle -\langle {u}_{i}\rangle \langle {u}_{j}\rangle$. The distribution of individual particle amplitudes also describes the volume of the beam in phase space.

The amplitude is defined by^30^

3 $${A}_{\perp }={\varepsilon }_{\perp }{R}^{2}({\bf{u}},\langle {\bf{u}}\rangle )$$

where *R*^2^(**u**, **v**) is the square of the distance between two points, **u** and **v**, in the phase space, normalized to the covariance matrix:

4 $${R}^{2}({\bf{u}},{\bf{v}})={({\bf{u}}-{\bf{v}})}^{{\rm{T}}}{V}^{-1}({\bf{u}}-{\bf{v}})$$

The normalized r.m.s. emittance is proportional to the mean of the particle amplitude distribution. In the approximation that particles travel near the beam axis, and in the absence of cooling, the particle amplitudes and the normalized r.m.s. emittance are conserved quantities. If the beam is well described by a multivariate Gaussian distribution, then *R*^2^ is distributed according to a *χ*^2^ distribution with four degrees of freedom, so the amplitudes are distributed according to

5 $$f({A}_{\perp })=\frac{{A}_{\perp }}{4{\varepsilon }_{\perp }^{2}}\exp \left(\frac{-{A}_{\perp }}{2{\varepsilon }_{\perp }}\right)$$

The rate of change of the normalized transverse emittance as the beam passes through an absorber is given approximately by^8,29,31^

6 $$\frac{{\rm{d}}{\varepsilon }_{\perp }}{{\rm{d}}z}\approx -\frac{{\varepsilon }_{\perp }}{{\beta }^{2}{E}_{\mu }}|\frac{{\rm{d}}{E}_{\mu }}{{\rm{d}}z}|+\frac{{\beta }_{\perp }{(13.6{\rm{M}}{\rm{e}}{\rm{V}}{c}^{-1})}^{2}}{2{\beta }^{3}{E}_{\mu }{m}_{\mu }{X}_{0}}$$

where *βc* is the muon velocity, *E*_*μ*_ is the muon energy, |d*E*_μ_/d*z*| is the mean energy loss per unit path length, *X*_0_ is the radiation length of the absorber and *β*_⊥_ is the transverse betatron function at the absorber^29^. The first term of this equation describes ‘cooling’ by ionization energy loss and the second term describes ‘heating’ by multiple Coulomb scattering. Equation (6) implies that there is an equilibrium emittance for which the emittance change is zero.

If the beam is well described by a multivariate Gaussian distribution both before and after cooling, then the downstream and upstream amplitude distributions *f*^ d^(*A*_⊥_) and *f*^ u^(*A*_⊥_) are related to the downstream and upstream emittances ${\varepsilon }_{\perp }^{{\rm{d}}}$ and ${\varepsilon }_{\perp }^{{\rm{u}}}$ by

7 $$\frac{{f}^{{\rm{d}}}({A}_{\perp })}{{f}^{{\rm{u}}}({A}_{\perp })}={\left(\frac{{\varepsilon }_{\perp }^{{\rm{u}}}}{{\varepsilon }_{\perp }^{{\rm{d}}}}\right)}^{2}\exp \left[-\frac{{A}_{\perp }}{2}\left(\frac{1}{{\varepsilon }_{\perp }^{{\rm{d}}}}-\frac{1}{{\varepsilon }_{\perp }^{{\rm{u}}}}\right)\right]$$

In the experiment described in this paper, many particles do not travel near the beam axis. These particles experience effects from optical aberrations, as well as geometrical effects such as scraping, in which high-amplitude particles outside the experiment’s aperture are removed from the beam. Scraping reduces the emittance of the ensemble and selectively removes those particles that scatter more than the rest of the ensemble. Optical aberrations and scraping introduce a bias in the change in r.m.s. emittance that occurs because of ionization cooling. In this work the distribution of amplitudes is studied. To expose the behaviour in the beam core, independently of aberrations affecting the beam tail, *V* and *ε*_⊥_ are recalculated for each amplitude bin, including particles that are in lower-amplitude bins and excluding particles that are in higher-amplitude bins. This results in a distribution that, in the core of the beam, is independent of scraping effects and spherical aberrations.

The change in phase-space density provides a direct measurement of the cooling effect. The *k*-nearest-neighbour algorithm provides a robust non-parametric estimator of the phase-space density of the muon ensemble^32,34,52^. The separation of pairs of muons is characterized by the normalized squared distance, ${R}_{ij}^{2}({{\bf{u}}}_{i},{{\bf{u}}}_{j})$, between muons with positions **u**_*i*_ and **u**_*j*_. A volume ${{\mathscr{V}}}_{ik}$ is associated with each particle, which corresponds to the hypersphere that is centred on **u**_*i*_ and intersects the *k*th nearest particle (that is, the particle that has the *k*th smallest *R*_*ij*_). The density, *ρ*_*i*_, associated with the *i*th particle is estimated by

8 $${\rho }_{i}({{\bf{u}}}_{i})=\frac{k}{n{|V|}^{1/2}}\frac{1}{{{\mathscr{V}}}_{ik}}=\frac{2k}{n{{\rm{\pi }}}^{2}{|V|}^{1/2}}\frac{1}{{R}_{ik}^{4}}$$

where *n* is the number of particles in the ensemble. An optimal value for *k* is used, $k={n}^{4/(4+d)}=\sqrt{n}$, with phase-space dimension *d* = 4 (ref. ^32^).

### Data taking and reconstruction

Data were buffered in the front-end electronics and read out after each target actuation. Data storage was triggered by a coincidence of signals in the photomultiplier tubes (PMTs) serving a single scintillator slab in the upstream TOF station closest to the cooling channel (TOF1). The data recorded in response to a particular trigger are referred to as a ‘particle event’.

Each TOF station was composed of a number of scintillator slabs that were read out using a pair of PMTs, one mounted at each end of each slab. The reconstruction of the data began with the search for coincidences in the signals from the two PMTs serving any one slab in a TOF plane. Such coincidences are referred to as ‘slab hits’. ‘Space points’ were then formed from the intersection of slab hits in the *x* and *y* projections of each TOF station separately. The position and time at which a particle giving rise to the space point crossed the TOF station were then calculated using the slab position and the times measured in each of the PMTs. The relative timing of the two upstream TOF stations (TOF0 and TOF1) was calibrated relative to the measured time taken for electrons to pass between the two TOF detectors, on the assumption that they travelled at the speed of light.

Signals in the tracker readout were collected to reconstruct the helical trajectories (‘tracks’) of charged particles in the upstream and downstream trackers (TKU and TKD, respectively). Multiple Coulomb scattering introduced significant uncertainties in the reconstruction of the helical trajectory of tracks with a bending radius of less than 5 mm. For this class of track, the momentum was deduced by combining the tracker measurement with the measurements from nearby detectors. The track-fitting quality was characterized by the *χ*^2^ per degree of freedom

9 $${\chi }_{{\rm{df}}}^{2}=\frac{1}{n}\sum _{i}\frac{{\rm{\delta }}{x}_{i}^{2}}{{\sigma }_{i}^{2}}$$

where δ*x*_*i*_ is the distance between the fitted track and the measured signal in the *i*th tracker plane, *σ*_*i*_ is the resolution of the position measurement in the tracker planes and *n* is the number of planes that had a signal used in the track reconstruction. Further details of the reconstruction and simulation may be found in ref. ^50^.

### Beam selection

Measurements made in the instrumentation upstream of the absorber were used to select the input beam. The input beam (the upstream sample) was composed of events that satisfied the following criteria:

- Exactly one space point was found in TOF0 and TOF1 and exactly one track in TKU.
- The track in TKU had ${\chi }_{{\rm{df}}}^{2} < 8$ and was contained within the 150-mm fiducial radius over the full length of TKU.
- The track in TKU had a reconstructed momentum in the range 135–145 MeV *c*^−1^, corresponding to the momentum acceptance of the cooling cell.
- The time-of-flight between TOF0 and TOF1 was consistent with that of a muon, given the momentum measured in TKU.
- The radius at which the track in TKU passed through the diffuser was smaller than the diffuser aperture.

The beam emerging from the cooling cell (the downstream sample) was characterized using the subset of the upstream sample that satisfied the following criteria:

- Exactly one track was found in TKD.
- The track in TKD had ${\chi }_{{\rm{df}}}^{2} < 8$ and was contained within the 150-mm fiducial radius of TKD over the full length of the tracker.

The same sample-selection criteria were used to select events from the simulation of the experiment, which included a reconstruction of the electronics signals expected for the simulated particles.

### Calculation of amplitudes

**Extended Data Fig. 1:**
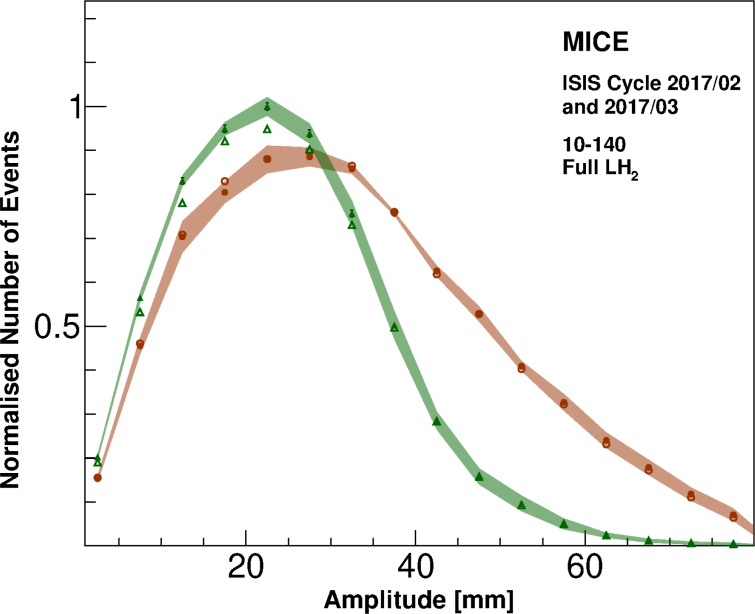
Corrected and uncorrected amplitude distributions for the 10–140 ‘LH2 full’ configuration. The uncorrected data are shown by open points and the corrected data by filled points. Orange circles correspond to the upstream distribution and green triangles to the downstream distribution. Shading represents the estimated total standard error. Error bars show the statistical error and for most points are smaller than the markers. Source Data

The amplitude distributions obtained from the upstream and downstream samples were corrected for the effects of the detector efficiency and resolution and for the migration of events between amplitude bins. The corrected number of events in a bin, ${N}_{i}^{{\rm{corr}}}$, was calculated from the raw number of events, ${N}_{j}^{{\rm{raw}}}$, using

10 $${N}_{i}^{{\rm{corr}}}={E}_{i}\sum _{j}{S}_{ij}{N}_{j}^{{\rm{raw}}}$$

where *E*_*i*_ is the efficiency correction factor and *S*_*ij*_ accounts for the detector resolution and event migration. *E*_*i*_ and *S*_*ij*_ were estimated from the simulation of the experiment. The uncorrected and corrected amplitude distributions for a particular configuration are shown in Extended Data Fig. 1. The correction is small relative to the ionization cooling effect, which is clear even in the uncorrected distributions.

It can be seen from equation (7) that in the limit of small amplitudes, and in the approximation that the beam is normally distributed in the phase-space variables, the ratio of the number of muons is equal to the ratio of the square of the emittances,

11 $$\mathop{\mathrm{lim}}\limits_{{A}_{\perp }\to 0}\frac{{f}^{{\rm{d}}}({A}_{\perp })}{{f}^{{\rm{u}}}({A}_{\perp })}={\left(\frac{{\varepsilon }_{\perp }^{{\rm{u}}}}{{\varepsilon }_{\perp }^{{\rm{d}}}}\right)}^{2}$$

The ratio of *f*^ d^ to *f*^ u^ in the lowest-amplitude bin of Fig. 3, which is an approximation to this ratio, is listed in Extended Data Table 1.

## Online content

Any methods, additional references, Nature Research reporting summaries, source data, extended data, supplementary information, acknowledgements, peer review information; details of author contributions and competing interests; and statements of data and code availability are available at 10.1038/s41586-020-1958-9.

## Source data

Source Data Fig. 3

Source Data Fig. 4

Source Data Fig. 5

Source Data Extended Data Fig. 1

## Figures and Tables

**Extended Data Table 1:**
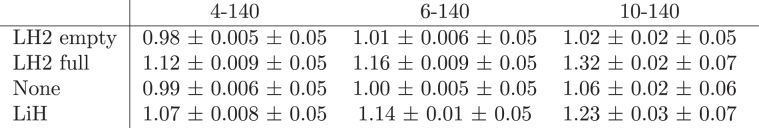
Ratio of number of muons downstream to number of muons upstream having an amplitude of less than 5 mm

Uncertainties denote standard error; statistical uncertainty is followed by the total uncertainty.

**Extended Data Table 2:**
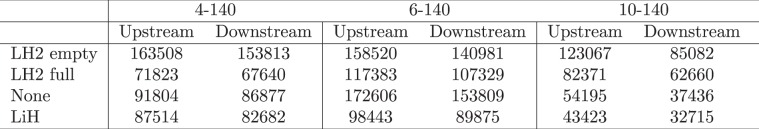
Number of events in the samples shown in Fig. 3–5

## Data Availability

The unprocessed and reconstructed data that support the findings of this study are publicly available on the GridPP computing Grid at 10.17633/rd.brunel.3179644 (MICE unprocessed data) and 10.17633/rd.brunel.5955850 (MICE reconstructed data). Source data for Figs. 3–5 and Extended Data Fig. 1 are provided with the paper. Publications using MICE data must contain the following statement: “We gratefully acknowledge the MICE collaboration for allowing us access to their data. Third-party results are not endorsed by the MICE collaboration.”

## Code availability

The MAUS software^50^ that was used to reconstruct and analyse the MICE data is available at 10.17633/rd.brunel.8337542. The analysis presented here used MAUS version 3.3.2.
